# Supplementary material for: Inhibition of the STAT3/Fanconi anemia axis is synthetic lethal with PARP inhibition in breast cancer
Source: Nat Commun. 2025 Mar 4;16:2159. doi: 10.1038/s41467-025-57476-4 (PMC11880418; doi:10.1038/s41467-025-57476-4)
Supplement: Supplementary file 1 — Supplementary Information [file 41467_2025_57476_MOESM1_ESM.pdf]

Supplementary Information of Inhibition of the STAT3/Fanconi anemia axis is synthetic lethal with PARP inhibition in breast cancer

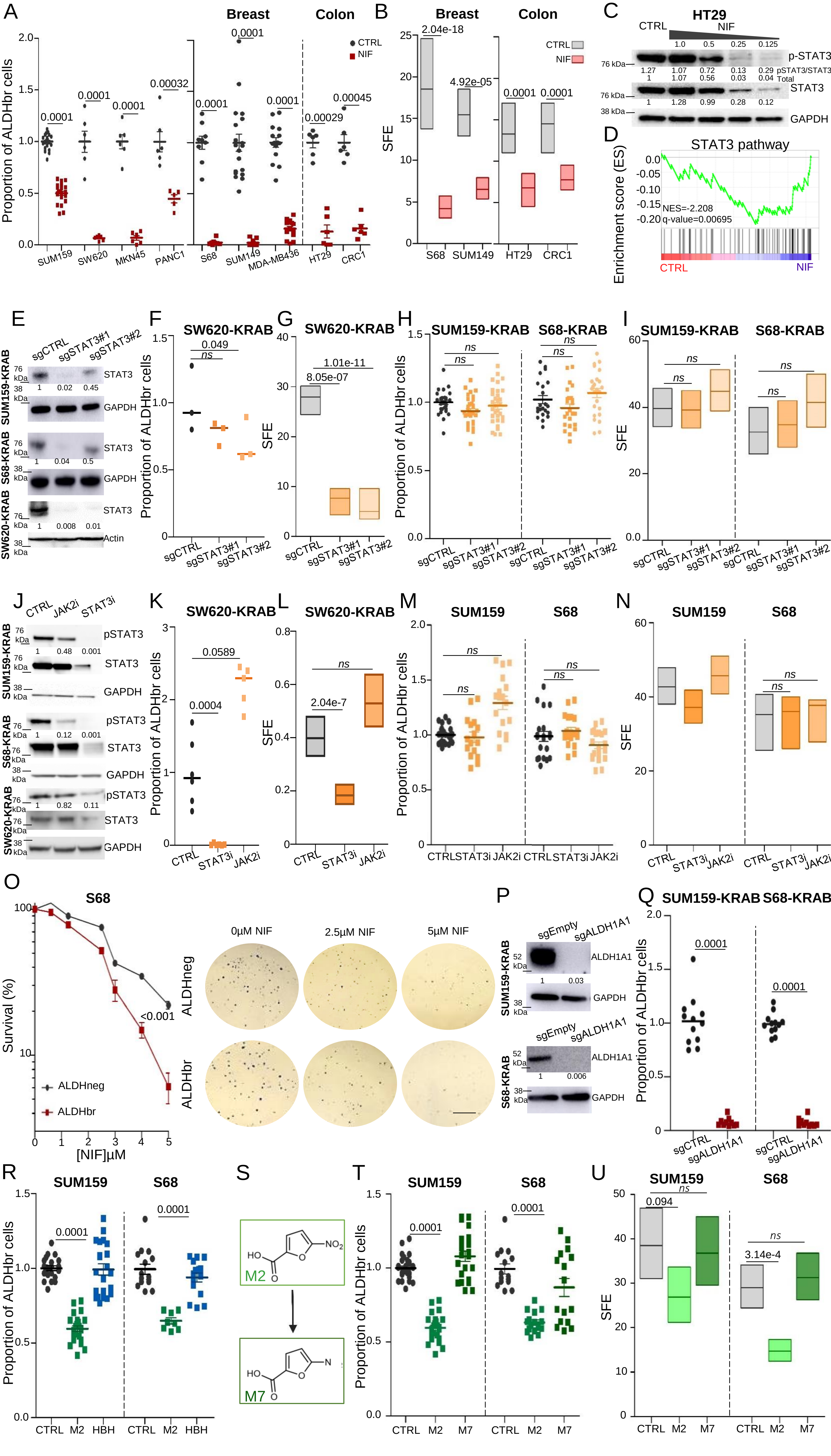
A
Proportion of ALDHbr cells
Breast
Colon
CTRL
NIF
SUM159
SW620
MKN45
PANC1
S68
SUM149
MDA-MB436
HT29
CRC1
B
SFE
2.04e-18
4.92e-05
C
HT29
p-STAT3
STAT3
GAPDH
pSTAT3/STAT3
Total
D
STAT3 pathway
Enrichment score (ES)
NES=-2.208
q-value=0.00695
E
sgCTRL
sgSTAT3#1
sgSTAT3#2
SUM159-KRAB
S68-KRAB
SW620-KRAB
Actin
F
SW620-KRAB
G
H
SUM159-KRAB
I
J
JAK2i
STAT3i
pSTAT3
K
L
M
SUM159
S68
N
O
Survival (%)
[NIF]µM
ALDHneg
ALDHbr
0µM NIF
2.5µM NIF
5µM NIF
P
sgEmpty
sgALDH1A1
ALDH1A1
Q
R
HBH
M2
S
M7
T
M7
U
Figure

Supplementary Figure 1. The anti-CSC compound Nifuroxazide is bioactivated by ALDH and inhibits STAT3 signalling. A. Proportion of ALDHbr cells following NIF treatment compared with the untreated condition (CTRL). **B.** SFE of breast and colon cell lines under treatment with NIF compared with the untreated condition (CTRL). (n=6). **C.** Western blot of STAT3 and p-STAT3 in NIF-treated HT29 colon cells compared to untreated conditions (CTRL). The mean intensities are indicated below each band for each condition from n=3 independent experiments. **D.** Pre-ranked GSEA interrogating differential expression between ALDHbr CTRL and NIF treated SUM159 and SW620 cells of genes involved in STAT3 pathway. **E**. Western blot of STAT3 SUM159-KRAB, S68-KRAB and SW620-KRAB with sgCTRL or sgSTAT3. The mean intensities are indicated below each band for each condition from n=3 independent experiments. **F,H**. Proportion of ALDHbr cells in colon (SW620-KRAB) and breast (SUM159-KRAB and S68-KRAB) cells with sgCTRL or sgSTAT3. **G,I**. SFE of colon (SW620-KRAB) and breast (SUM159-KRAB, S68-KRAB) cells with sgCTRL or sgSTAT3. **J.** Western blot of p-STAT3 and STAT3 SUM159-KRAB, S68-KRAB and SW620-KRAB following 24H of STAT3i or JAK2i treatment. The mean intensities are indicated below each band for each condition from n=3 independent experiments. **K,M**. Proportion of ALDHbr cells following STAT3i and JAK2i treatment compared with the untreated condition (CTRL). **L,N**.SFE of SW620, SUM159 and S68 under STAT3i and JAK2i treatment. **O**. S68 cells exposed to various concentrations of NIF were subjected to clonogenic survival assays and representative images (right panel). Scale bar: 1 cm. **P**. Western blot of ALDH1A1 in SUM159-KRAB and S68-KRAB with sgEmpty and sgALDH1A1 constructs. The mean intensities are indicated below each band for each condition from n=3 independent experiments. **Q.** Proportion of ALDHbr cells in SUM159-KRAB and S68-KRAB with sgEmpty or sgALDH1A1. **R,T**. Proportion of ALDHbr cells exposed to the M2, HBH or M7 treatments compared to untreated conditions (CTRL). **S**. Chemical structure of M2 and M7 metabolites. **U**. SFE of SUM159 and S68 exposed to M2 or M7 treatment compared to the untreated condition (CTRL). In (**B**, **G**, **I**, **L**, **N**, **U**) box represents mean ± margin of error (95% Confidence Interval) of n=3 independent experiments. Statistical significance was calculated using one-sided Chi-squared test or t-test. In (**A**, **F**, **H**, **K**, **M**, **Q**, **R**, **T**) data are shown as mean +/- SEM (Standard Error of Mean) of n=3 independent experiments, according to two-way ANOVA followed by Sidak multiple range test. ns (not significant). Source data are provided as a Source Data file.

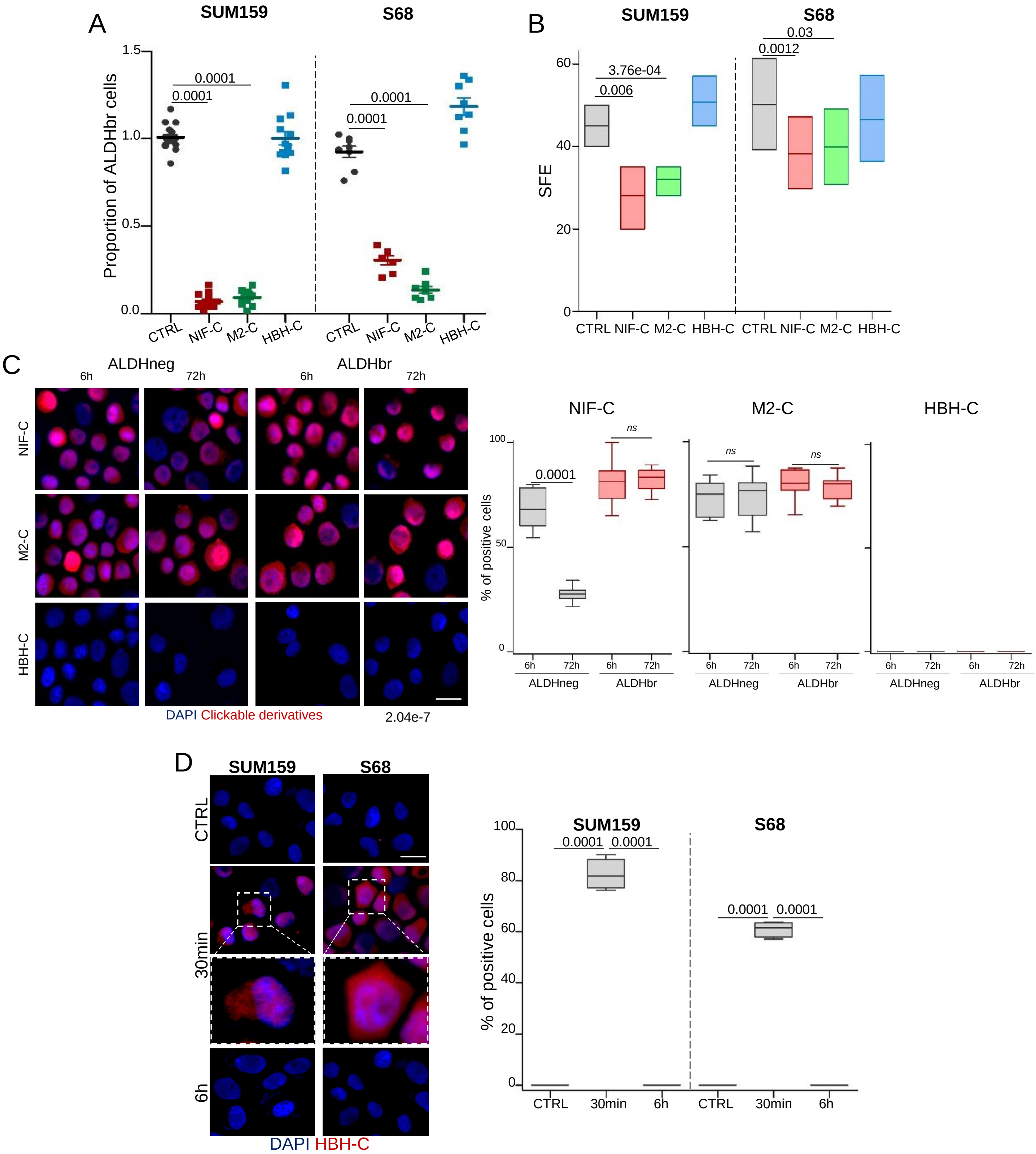
A
SUM159
S68
Proportion of ALDHbr cells
1.5
1.0
0.5
0.0
0.0001
0.0001
0.0001
0.0001
CTRL
NIF-C
M2-C
HBH-C
CTRL
NIF-C
M2-C
HBH-C
B
SUM159
S68
SFE
60
40
20
0
3.76e-04
0.006
0.03
0.0012
CTRL NIF-C M2-C HBH-C CTRL NIF-C M2-C HBH-C
C
ALDHneg
ALDHbr
6h
72h
6h
72h
NIF-C
M2-C
HBH-C
DAPI Clickable derivatives
2.04e-7
NIF-C
M2-C
HBH-C
% of positive cells
100
50
0
0.0001
ns
ns
ns
6h
72h
6h
72h
ALDHneg
ALDHbr
D
SUM159
S68
CTRL
30min
6h
DAPI HBH-C
SUM159
S68
% of positive cells
100
80
60
40
20
0
0.0001
0.0001
0.0001
0.0001
CTRL
30min
6h
CTRL
30min
6h

Supplementary Figure 2. NIF clickable analogs effect on CSC and in situ detection. A. Proportion of ALDHbr cells following click compounds treatment compared with the untreated condition (CTRL). **B**. SFE of SUM159 and S68 after click compounds treatment compared to the untreated conditions (CTRL). **C**. Detection of NIF-C, M2-C and HBH-C (red staining) in ALDHneg and ALDHbr S68 cells after 6 and 72 hours of treatment. Nuclei are counterstained with DAPI (blue staining). Scale bar: 3.5 μm. Box plot represent the proportion of cell with positive click-labelling after different time of treatment. **D**. Detection of HBH-C (red staining) in SUM159 and S68 cells after 30minutes and 6 hours of treatment. Nuclei are counterstained with DAPI (blue staining). Third line represents an enlargement of the area delimited by dashed line. Scale bar: 3.5 μm. Box plot represents the proportion of cell presenting a positive click- after different times of treatment. In (**A, C-D**) boxplots represent median and quartile and whiskers minimum to maximum. In (**B**) box represents mean ± margin of error (95% Confidence Interval) of n=3 independent experiments. ns (not significant), p-value estimated according to one-sided Fisher test (**A**), One-sided chi-squared test (**B**) and one-way ANOVA followed by Dunn's multiple comparison test (**C-D**). Source data are provided as a Source Data file.

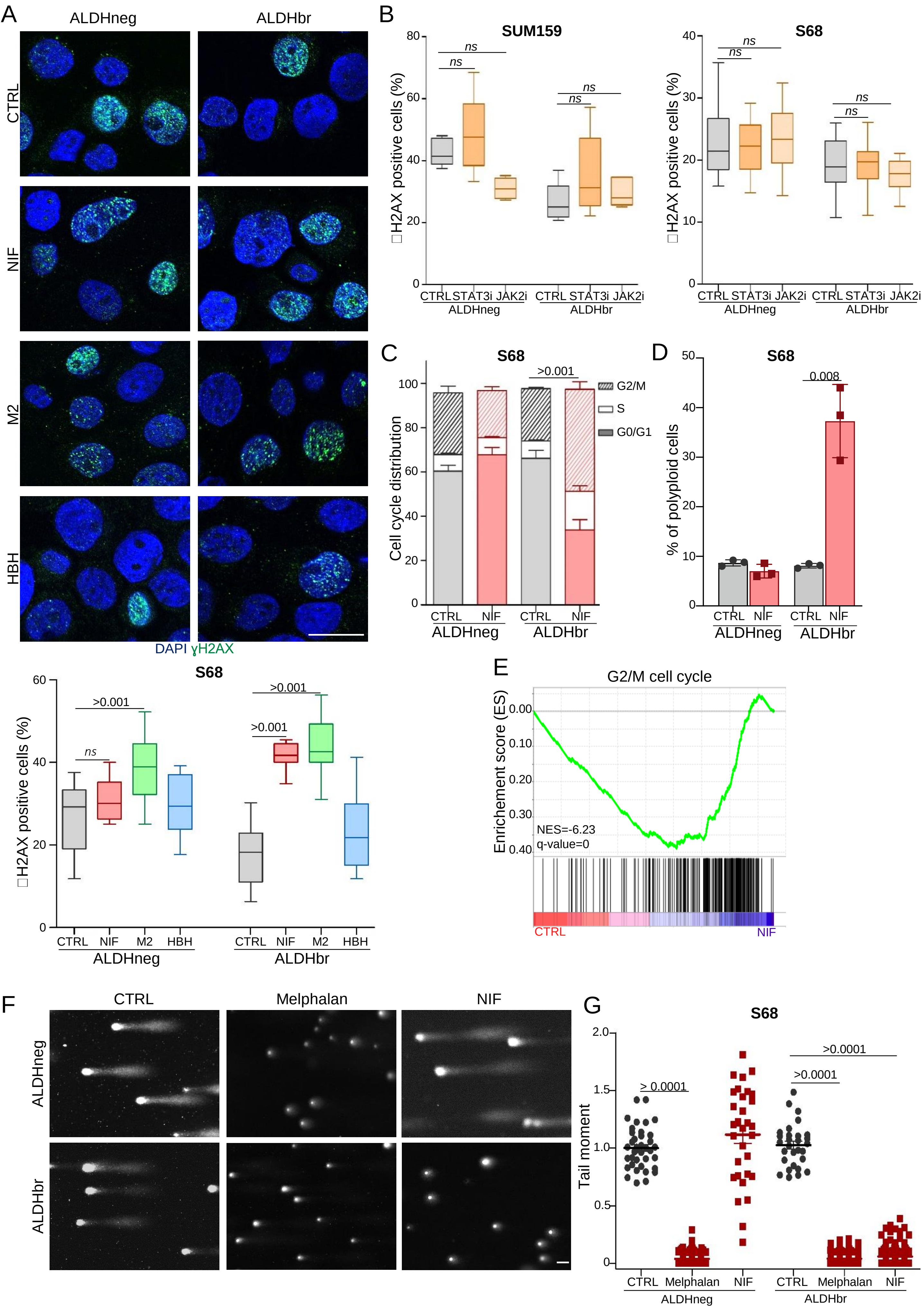
A
ALDHneg
ALDHbr
CTRL
NIF
M2
HBH
DAPI γH2AX
B
SUM159
S68
γH2AX positive cells (%)
ns
CTRL STAT3i JAK2i
ALDHneg
ALDHbr
C
S68
Cell cycle distribution
>0.001
G2/M
S
G0/G1
CTRL NIF
D
% of polyploid cells
0.008
E
G2/M cell cycle
Enrichement score (ES)
NES=-6.23
q-value=0
CTRL
NIF
γH2AX positive cells (%)
>0.001
CTRL NIF M2 HBH
F
CTRL
Melphalan
NIF
G
S68
Tail moment
> 0.0001
>0.0001
CTRL Melphalan NIF

Supplementary Figure 3. NIF treatment effect on DNA integrity and cell cycle progression A. Representative images of γH2AX foci (green staining) in ALDHneg and ALDHbr S68 cells after NIF and M2 treatment compared to the untreated conditions (CTRL). Nuclei are counterstained with DAPI (blue staining). Scale bar: 15μm. Box plots (bottom panel) represent the proportion of γH2AX -positive cells (median and quartiles) with whiskers (minimum to maximum), for each cell subpopulation under the indicated treatment. (n=4). **B**. Box plot represents the proportion of γH2AX -positive cells in SUM159 and S68 for each cell subpopulations (ALDHneg and ALDHbr) after 3 days of STAT3 inhibitor (STAT3i) or JAK2 inhibitor (JAK2i) treatment. Box plot show (median and quartiles) with whiskers (minimum to maximum). **C-D**. Monitoring of cell cycle distribution by QIBC in ALDHneg or ALDHbr S68 cells exposed to NIF (C). Quantification of the proportion of polyploid cells (D). Data are presented as mean values +/-SD. **E**. GSEA interrogating differential expression between ALDHbr CTRL and NIF-treated SUM159 cells of genes involved in G2/M cell cycle signalling. **F-G**. Representative images of reverse comet assays conducted in ALDHbr and ALDHneg S68 cells subjected to the indicated treatments (F). Reverse comet assay quantification each dot corresponding to tail moment of one comet with the indicated drugs. Mean and SD are indicated (G). In (**A, B**) boxplots represent median and quartile and whiskers minimum to maximum. In (**C-D, G**) data are shown as mean +/- SEM of n=3 independent experiments. ns (not significant), p-value estimated according to one-way ANOVA followed by Dunn's multiple comparison test (**A, B**), or one-sided Fisher test (**C-D, G)**. Source data are provided as a Source Data file.

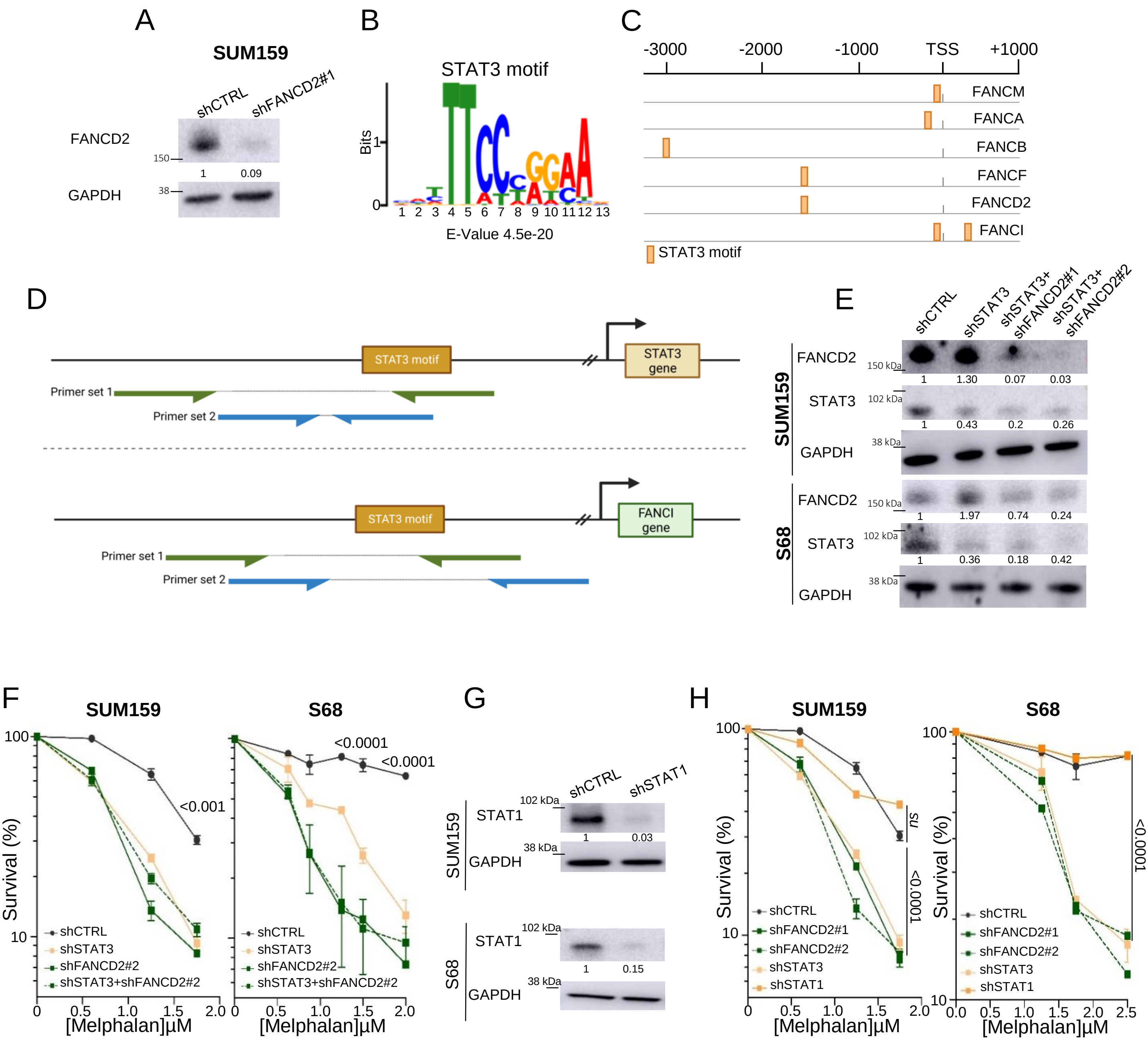
A
SUM159
shCTRL
shFANCD2#1
FANCD2
GAPDH
150
38
1
0.09
B
STAT3 motif
Bits
E-Value 4.5e-20
C
-3000
-2000
-1000
TSS
+1000
FANCM
FANCA
FANCB
FANCF
FANCD2
FANCI
STAT3 motif
D
STAT3 motif
STAT3 gene
Primer set 1
Primer set 2
FANCI gene
E
shCTRL
shSTAT3
shSTAT3+ shFANCD2#1
shSTAT3+ shFANCD2#2
SUM159
S68
FANCD2
STAT3
GAPDH
150 kDa
102 kDa
38 kDa
1 1.30 0.07 0.03
1 0.43 0.2 0.26
1 1.97 0.74 0.24
1 0.36 0.18 0.42
F
SUM159
S68
Survival (%)
[Melphalan]µM
<0.001
<0.0001
<0.0001
shCTRL
shSTAT3
shFANCD2#2
shSTAT3+shFANCD2#2
G
shCTRL
shSTAT1
SUM159
S68
STAT1
GAPDH
102 kDa
38 kDa
1 0.03
1 0.15
H
SUM159
S68
Survival (%)
[Melphalan]µM
ns
<0.0001
<0.0001
shCTRL
shFANCD2#1
shFANCD2#2
shSTAT3
shSTAT1

Supplementary Figure 4. STAT3 and Fanconi anemia pathways interaction. A. Western blot of FANCD2 in shCTRL and shFANCD2#1 SUM159. The mean intensities are indicated below each band for each condition from n=3 independent experiments. **B-C**. STAT3 motif enrichment in FA pathway genes promoter (B) and its location on FA pathway genes promoter (C). **D**. Location on DNA sequence of STAT3 and FANCI primer set used for CUT&RUN-qPCR analysis. **E**. Detection of FANCD2 and STAT3 protein expression in shCTRL, shFANCD2 and shSTAT3 SUM159 and S68 cell protein extracts, using western blot. The mean intensities are indicated below each band for each condition from n=3 independent experiments. **F**. SUM159 and S68 shCTRL, shFANCD2#2 and shSTAT3 cells exposed to various concentrations of melphalan were subjected to clonogenic survival assays. **G**. Detection of STAT1 protein expression in shCTRL and shSTAT1 SUM159 and S68 cell protein extracts. The mean intensities are indicated below each band for each condition from n=3 independent experiments. **H**. SUM159 and S68 shCTRL, shFANCD2, shSTAT3 shFANCD2#1 or #2 and shSTAT1 cells exposed to various concentrations of melphalan were subjected to clonogenic survival assays. In (**F, H**) data are shown as mean +/- SEM of n=3 independent experiments. ns (not significant), p-value estimated according to two-way ANOVA followed by sidak multiple range test. Source data are provided as a Source Data file.

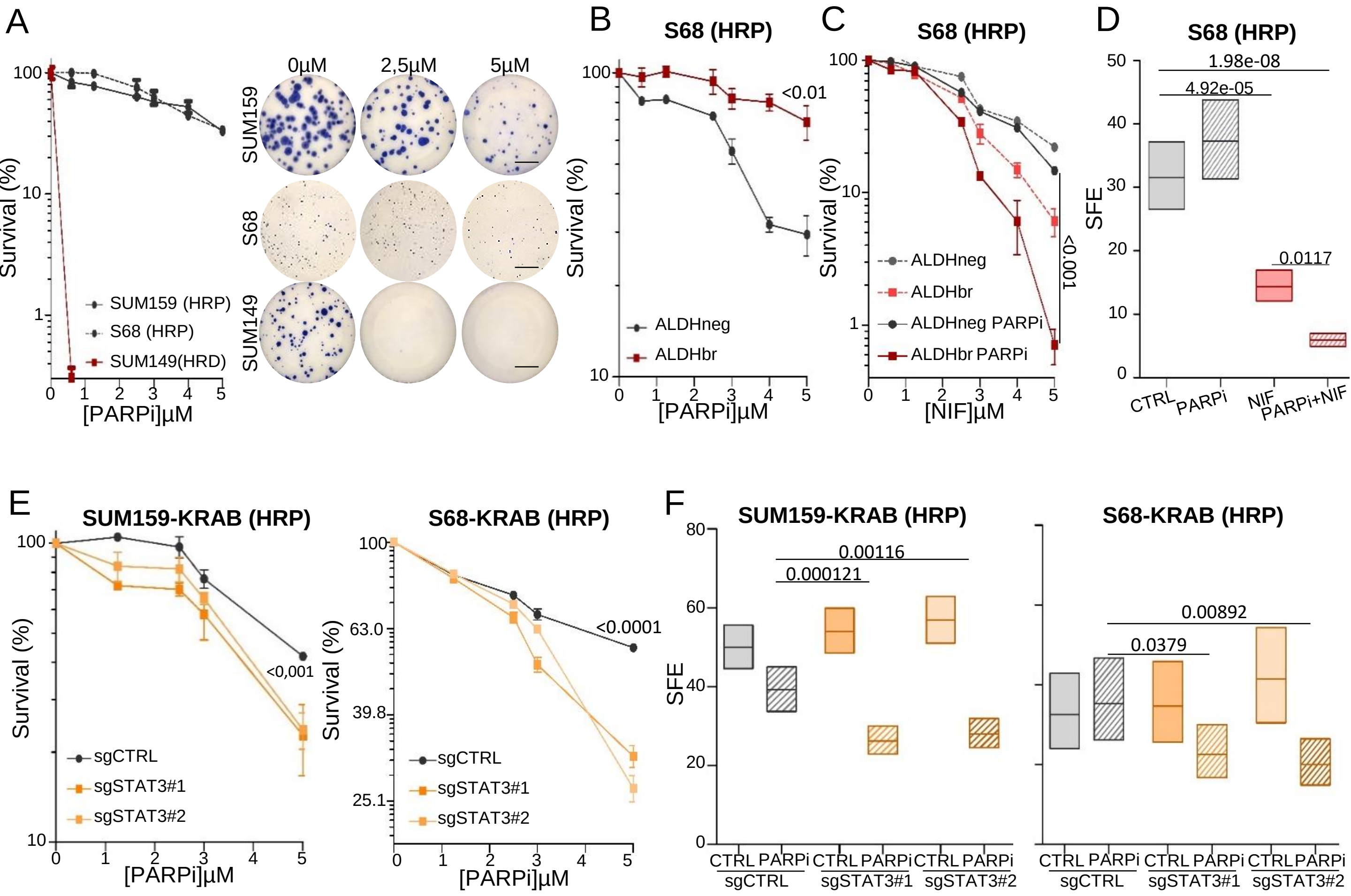
A
Survival (%)
100
10
1
SUM159 (HRP)
S68 (HRP)
SUM149(HRD)
[PARPi]µM
0µM
2,5µM
5µM
SUM159
S68
SUM149
B
S68 (HRP)
Survival (%)
<0.01
ALDHneg
ALDHbr
[PARPi]µM
C
S68 (HRP)
ALDHneg
ALDHbr
ALDHneg PARPi
ALDHbr PARPi
<0.001
[NIF]µM
D
S68 (HRP)
SFE
1.98e-08
4.92e-05
0.0117
CTRL
PARPi
NIF
PARPi+NIF
E
SUM159-KRAB (HRP)
S68-KRAB (HRP)
<0,001
<0.0001
sgCTRL
sgSTAT3#1
sgSTAT3#2
63.0
39.8
25.1
[PARPi]µM
F
SUM159-KRAB (HRP)
S68-KRAB (HRP)
0.00116
0.000121
0.00892
0.0379
CTRL
PARPi
sgCTRL
sgSTAT3#1
sgSTAT3#2

Supplementary Figure 5. Identification of synthetic lethality interaction between STAT3 and PARP inhibitions. A. SUM159, S68 and SUM149 cells exposed to various concentrations of PARPi were subjected to clonogenic survival assays each dot represent a mean value with SD (left panel), and representative images (right panel). Scal bar: 2.5cm for SUM159 and SUM149, 1.25cm for S68. **B-C**. S68 (HRP) ALDHneg and ALDHbr cells exposed to various concentrations of PARPi were subjected to clonogenic survival assays (**C**). S68 ALDHneg and ALDHbr cells treated with a sublethal dose of PARPi (or untreated cells) were exposed to various concentrations of NIF and subjected to clonogenic survival assays (**D**). Each dot represents a mean value with SD. **D**. SFE of S68 in NIF, PARPi on combination-treated conditions. , The box represents the confidence interval (mean and the maximum and minimum values) of n=3 independent experiments. **E**. SUM159-KRAB and S68-KRAB cells with sgCTRL or sgSTAT3 exposed to various concentrations of PARPi were subjected to clonogenic survival assays; each dot represents a mean value with SD. **F**. SFE of SUM159-KRAB and S68-KRAB cells with sgCTRL or sgSTAT3 and treated to PARPi. The box represents the confidence interval (mean and the maximum and minimum values) of n=3 independent experiments. In (**A-B, E**) data are shown as mean +/- SD of n=3 independent experiments and in (**D, F**) box represents mean ± margin of error (95% Confidence Interval) of n=3 independent experiments. ns (not significant), p-value estimated according to and one-way ANOVA followed by Dunn's multiple comparison test (**A-B, E**) and one-sided chi-squared test (**D, F)**. Source data are provided as a Source Data file.

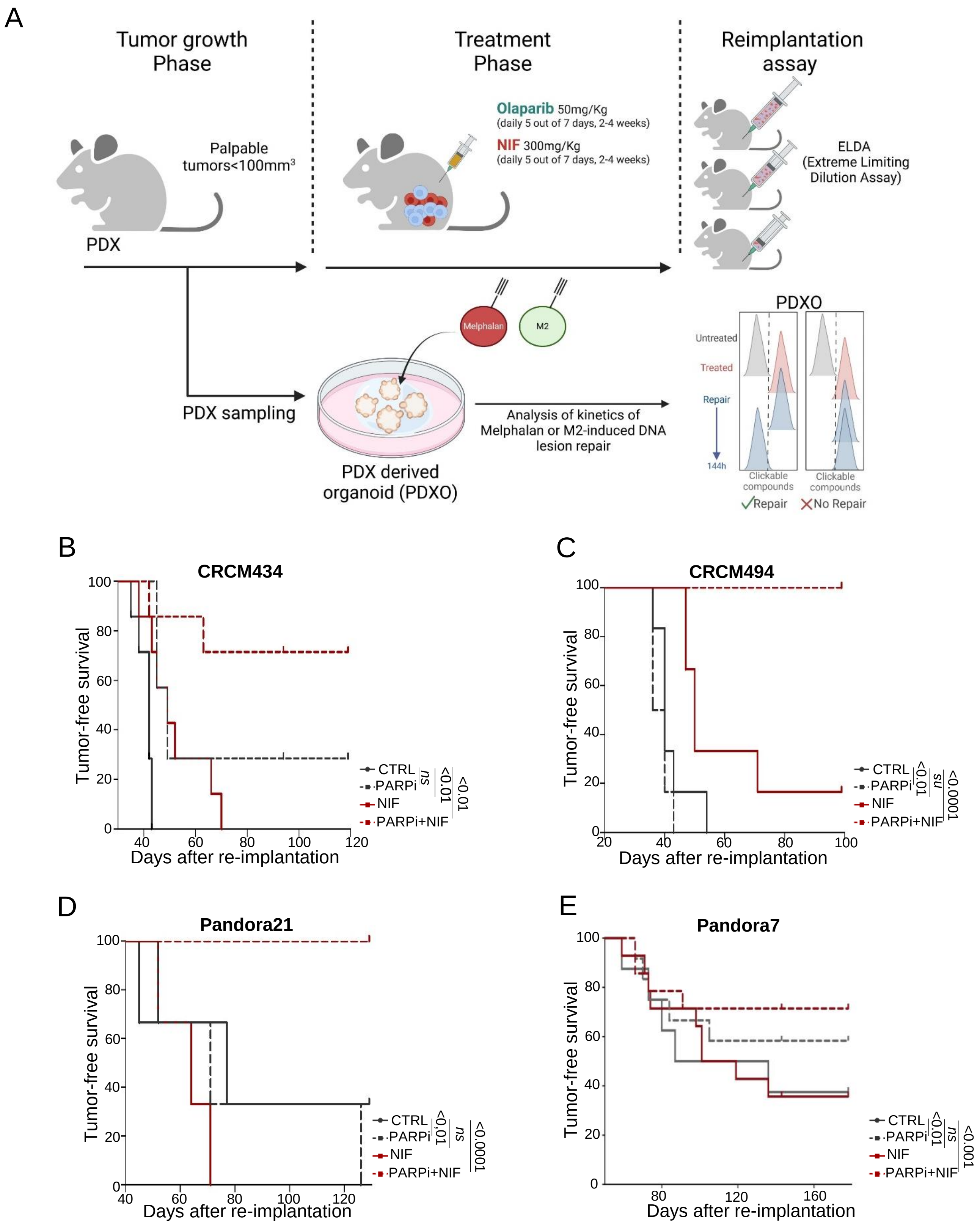

A
Tumor growth Phase
Treatment Phase
Reimplantation assay
Olaparib 50mg/Kg
(daily 5 out of 7 days, 2-4 weeks)
NIF 300mg/Kg
(daily 5 out of 7 days, 2-4 weeks)
Palpable tumors<100mm³
PDX
ELDA
(Extreme Limiting Dilution Assay)
Melphalan
M2
PDXO
Untreated
Treated
Repair
144h
PDX sampling
PDX derived organoid (PDXO)
Analysis of kinetics of Melphalan or M2-induced DNA lesion repair
Clickable compounds
Repair
No Repair
B
CRCM434
Tumor-free survival
Days after re-implantation
CTRL
PARPi
NIF
PARPi+NIF
ns
<0.01
<0.01
C
CRCM494
<0.01
ns
<0.0001
D
Pandora21
<0.01
ns
<0.0001
E
Pandora7
<0.01
ns
<0.001

Supplementary Figure 6. Preclinal assay testing the effect of Olaparib and Nifuroxazide treatment on breast tumor relapse capacity. A. Schematic representation of the in vivo experimental design. Created with BioRender.com. https://BioRender.com/b62d917. **B-E**. Kaplan-Meier tumor-free survival curves of mice xenografted with 5-100 residual cells from treated tumors for each PDX model (n=8). ns (not significant), p-value estimated according to Log-rank (Mantel-Cox) test. Source data are provided as a Source Data file.

A

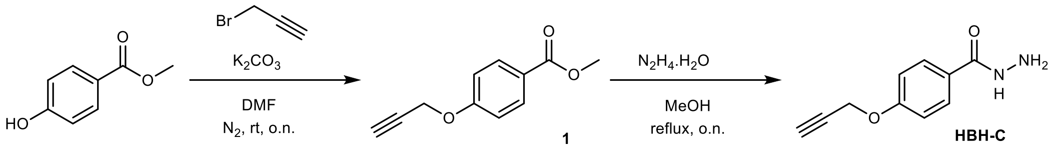
Br
K_2CO_3
DMF
N_2, rt, o.n.
$N_2H_4.H_2O$
MeOH
reflux, o.n.
HO
O
1
HBH-C
NH_2
N
H

B

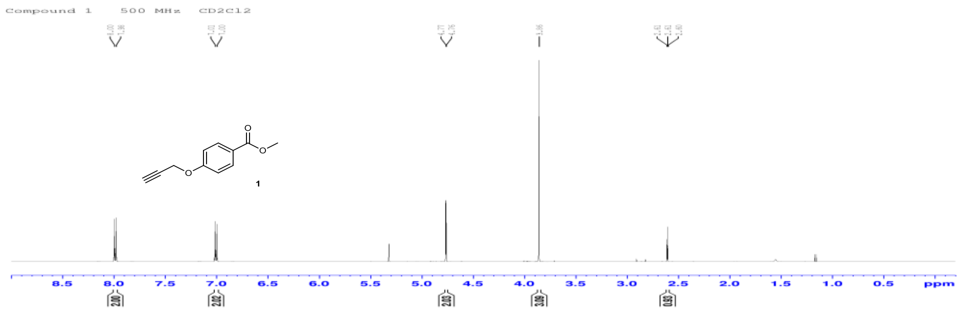
Compound 1 500 MHz CD2Cl2
1
ppm

C

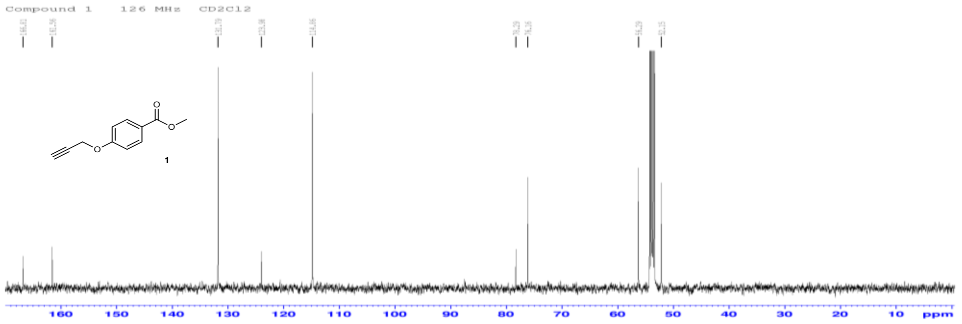
Compound 1 126 MHz CD2Cl2
1
ppm

D

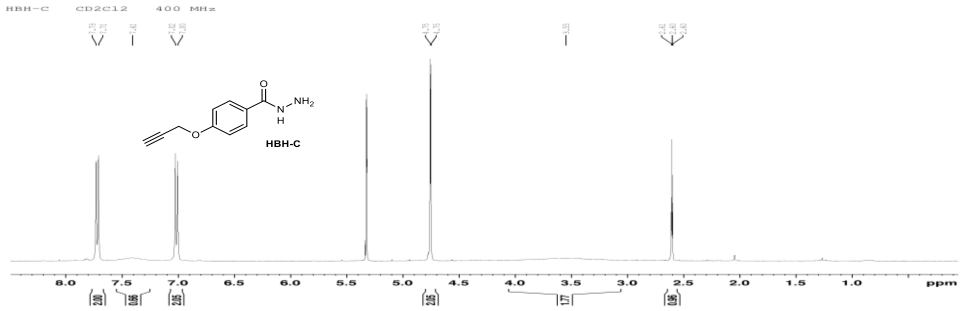
HBH-C CD2Cl2 400 MHz
HBH-C
ppm

E

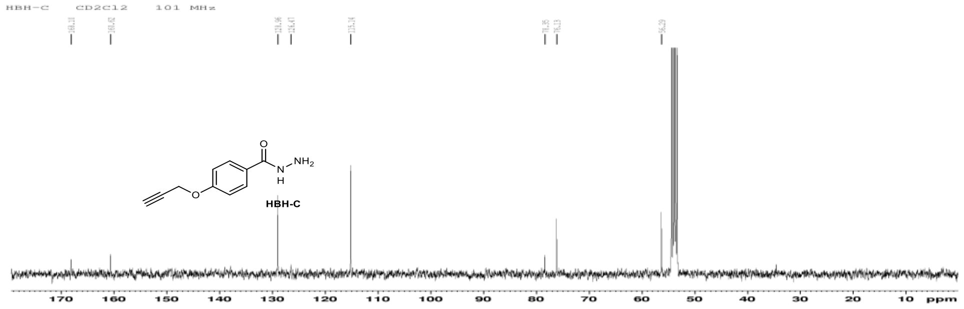
HBH-C CD2Cl2 101 MHz
HBH-C
ppm

F

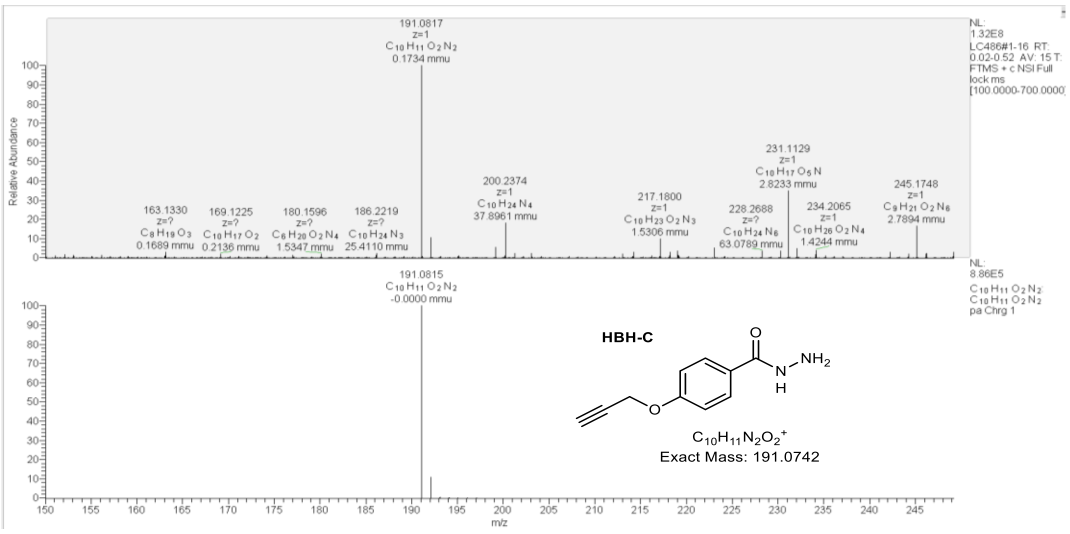
191.0817
z=1
$C_{10}H_{11}O_2N_2$
0.1734 mmu
Relative Abundance
NL:
1.32E8
LC486#1-16 RT:
FTMS + c NSI Full
191.0815
$C_{10}H_{11}O_2N_2$
-0.0000 mmu
NL:
8.86E5
pa Chrg 1
HBH-C
$C_{10}H_{11}N_2O_2^+$
Exact Mass: 191.0742
m/z

Supplementary Figure 7. 5-Spectroscopic and spectrometric analysis of synthesized compound 1 and HBH-C. A. Clickable HBH analog HBH-C procedure synthesis. **B**. ^{1}H NMR spectrum of compound 1 in dichloromethane-d2. Yield: 370 mg, 97%. Isolated as a pale brownish solid, >95% pure by NMR and a single spot by TLC; R_f: 0.63 in n-Hex/EtOAc, 80/20. ^{1}H NMR (500 MHz, CD_2Cl_2) δ ppm = 7.99 (d, *J* = 8.1 Hz, 2H), 7.00 (d, *J* = 8.1 Hz, 2H), 4.76 (d, *J* = 2.4 Hz, 2H), 3.86 (s, 3H), 2.61 (t, *J* = 2.4 Hz, 1H). **C**. ^{13}C NMR spectrum of compound 1 in dichloromethane-d2. ^{13}C NMR (126 MHz, CD_2Cl_2) δ ppm = 166.8; 161.6; 131.8 (2C); 124.0; 114.9 (2C); 78.3; 76.2; 56.3; 52.2. UPLC: R_T: 1.33 (classic system). **D**. ^{1}H NMR spectrum of HBH-C in dichloromethane-d2. Yield: 148 mg, 74%. Isolated as a white powder, >95% pure by NMR; ^{1}H NMR (400 MHz, CD_2Cl_2) δ ppm = 7.71 (d, *J* = 8.5 Hz, 2H), 7.40 (brs, 1H), 7.01 (d, *J* = 8.9 Hz, 2H), 4.75 (d, *J* = 2.5 Hz, 2H), 3.55 (brs, 2H), 2.60 (t, *J* = 2.5 Hz, 1H). **E**. ^{13}C NMR spectrum of HBH-C in dichloromethane-d2. ^{13}C NMR (101 MHz, CD_2Cl_2) δ ppm = 168.1; 160.6; 128.9 (2C); 126.5; 115.1 (2C); 78.4; 76.1; 56.3. UPLC: R_T: 1.36 (classic system). **F**. HRMS spectrum of HBH-C. HRMS (ESI^+) m/z $[M+H]^+$ Calcd for $C_{1O}H_{11}N_2O_2^+$ 191.0742; Found 191.0817. Source data are provided as a Source Data file.

A

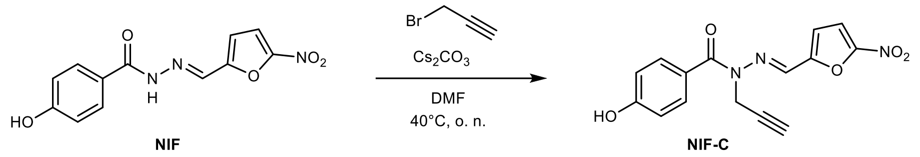

Br
Cs_2CO_3
DMF
40°C, o. n.
NIF
NIF-C
HO
NO_2

B

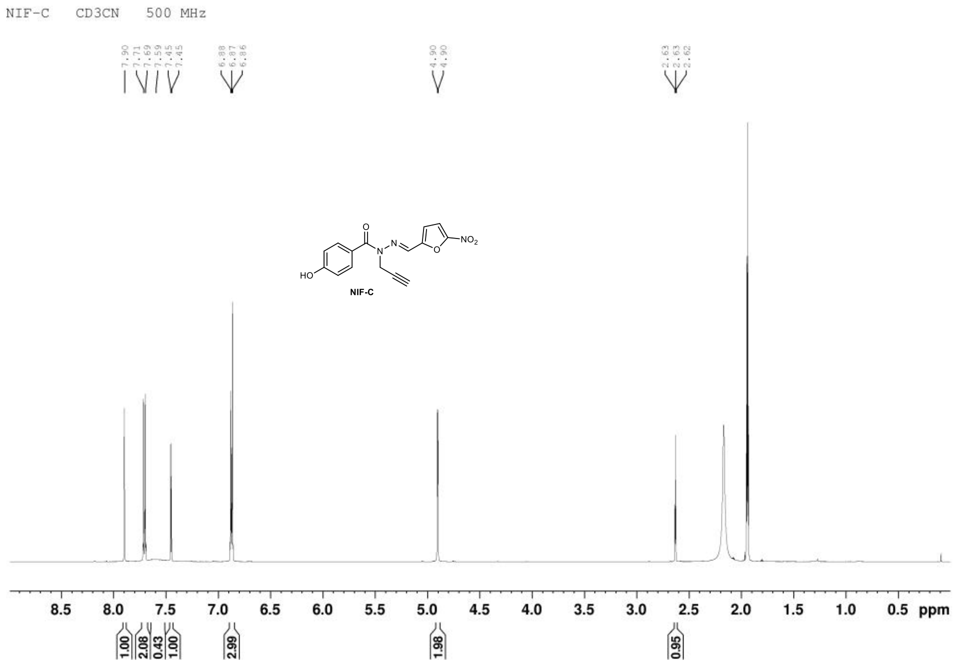

NIF-C CD3CN 500 MHz
7.90 7.71 7.69 7.59 7.45 7.45
6.88 6.87 6.86
4.90 4.90
2.63 2.63 2.62
NIF-C
1.00 2.08 0.43 1.00
2.99
1.98
0.95
ppm

C

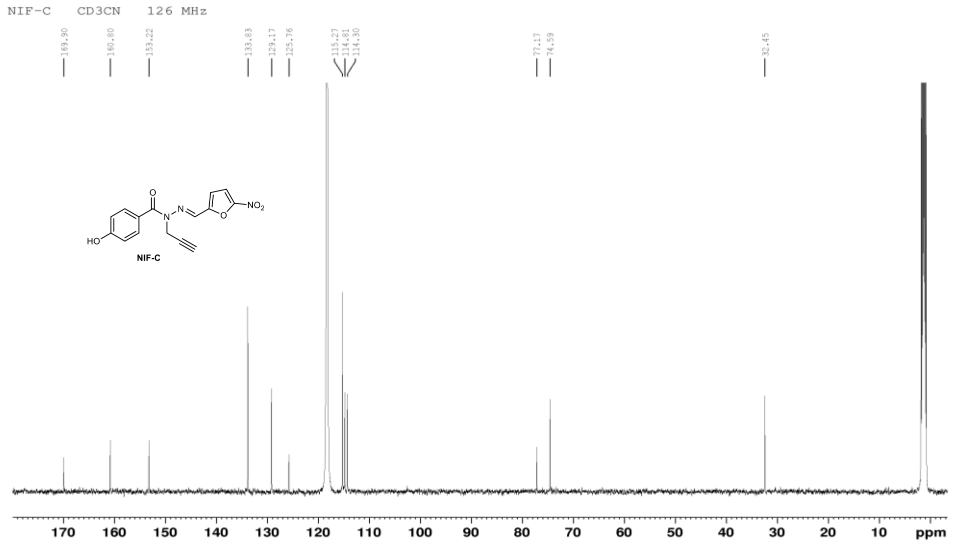

NIF-C CD3CN 126 MHz
169.90
160.80
153.22
133.83
129.17
125.76
115.27 114.81 114.30
77.17 74.59
32.45
NIF-C
ppm

D

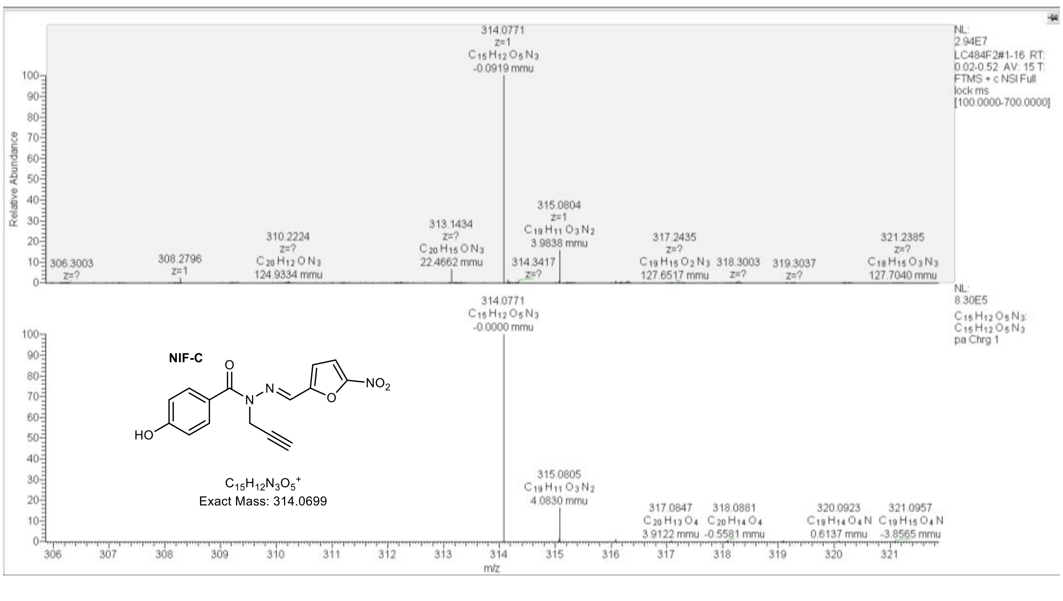

314.0771
z=1
$C_{15}H_{12}O_5N_3$
-0.0919 mmu
315.0804
z=1
$C_{19}H_{11}O_3N_2$
3.9838 mmu
NL:
2.94E7
LC484F2#1-16 RT: 0.02-0.52 AV: 15 T: FTMS + c NSI Full lock ms [100.0000-700.0000]
Relative Abundance
314.0771
$C_{15}H_{12}O_5N_3$
-0.0000 mmu
NL:
8.30E5
$C_{15}H_{12}O_5N_3$:
$C_{15}H_{12}O_5N_3$
pa Chrg 1
NIF-C
$C_{15}H_{12}N_3O_5^+$
Exact Mass: 314.0699
315.0805
$C_{19}H_{11}O_3N_2$
4.0830 mmu
m/z

Supplementary Figure 8. 5-Spectroscopic and spectrometric analysis of synthesized NIF-C. A. Clickable NIF analog NIF-C procedure synthesis. **B**. ^{1}H NMR spectrum of NIF-C in dichloromethane-d2. Yield: 15 mg, 9.6%. Isolated as a yellow powder, >95% pure by NMR and a single spot by TLC; R_f: 0.72 in n-Hex/EtOAc, 20/80. ^{1}H NMR (500 MHz, CD_3CN) δ ppm = 7.89 (s, 1H), 7.70 (d, *J* = 8.8 Hz, 2H), 7.45 (d, *J* = 3.9 Hz, 1H), 6.87 (m, 3H), 4.90 (d, *J* = 2.7 Hz, 2H), 2.63 (t, *J* = 2.6 Hz, 1H). **C**. ^{13}C NMR spectrum of NIF-C in dichloromethane-d2. ^{13}C NMR (126 MHz, CD_3CN) δ ppm = 169.9; 160.8; 153.2 (2C) ; 133.8 (2C) ; 129.2 ; 125.8 ; 115.3 (2C) ; 114.8; 114.3 ; 77.2; 74.6; 32.5. UPLC: R_T: 2.08 (classic system). **D**. HRMS spectrum of NIF-C. HRMS (ESI$^+$) m/z $[M+H]^+$ Calcd for $C_{15}H_{12}N_3O_5^+$ 314.0699; Found 314.0771. Source data are provided as a Source Data file.

A

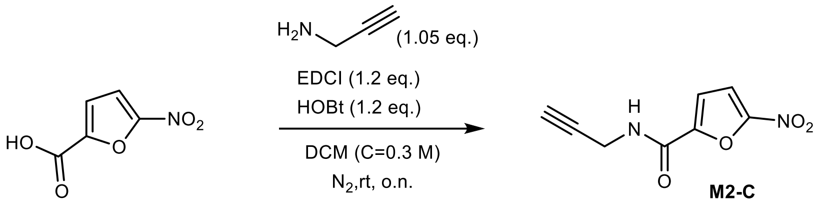

H2N
(1.05 eq.)
EDCI (1.2 eq.)
HOBt (1.2 eq.)
DCM (C=0.3 M)
N2,rt, o.n.
HO
O
NO2
H
N
M2-C

B

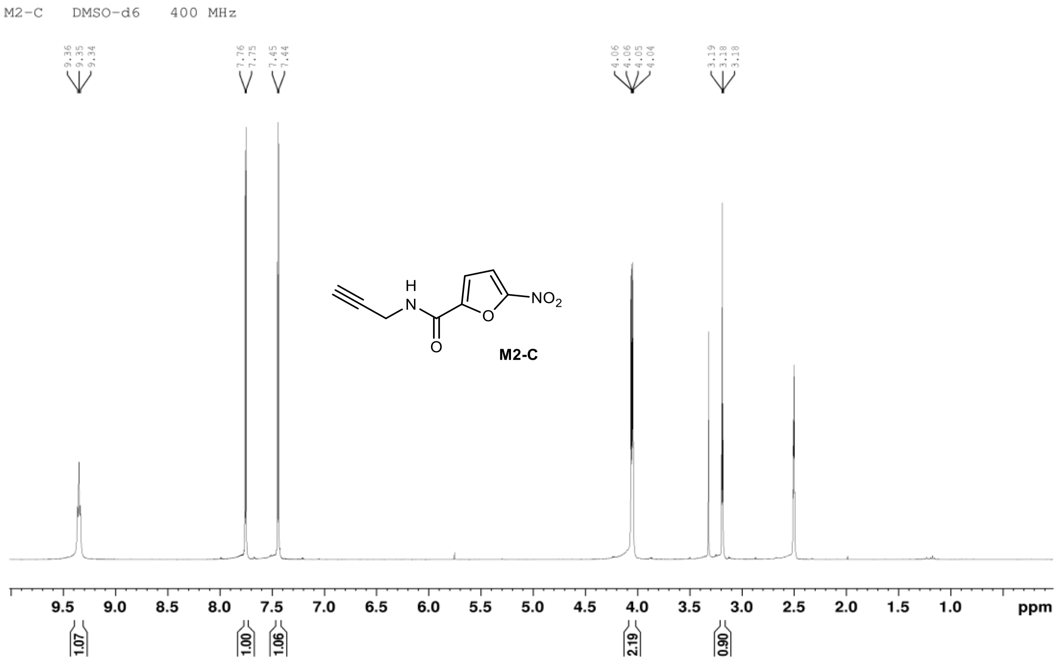

M2-C DMSO-d6 400 MHz
9.36 9.35 9.34
7.76 7.75
7.45 7.44
4.06 4.06 4.05 4.04
3.19 3.18 3.18
M2-C
9.5 9.0 8.5 8.0 7.5 7.0 6.5 6.0 5.5 5.0 4.5 4.0 3.5 3.0 2.5 2.0 1.5 1.0 ppm
1.07
1.00
1.06
2.19
0.90

C

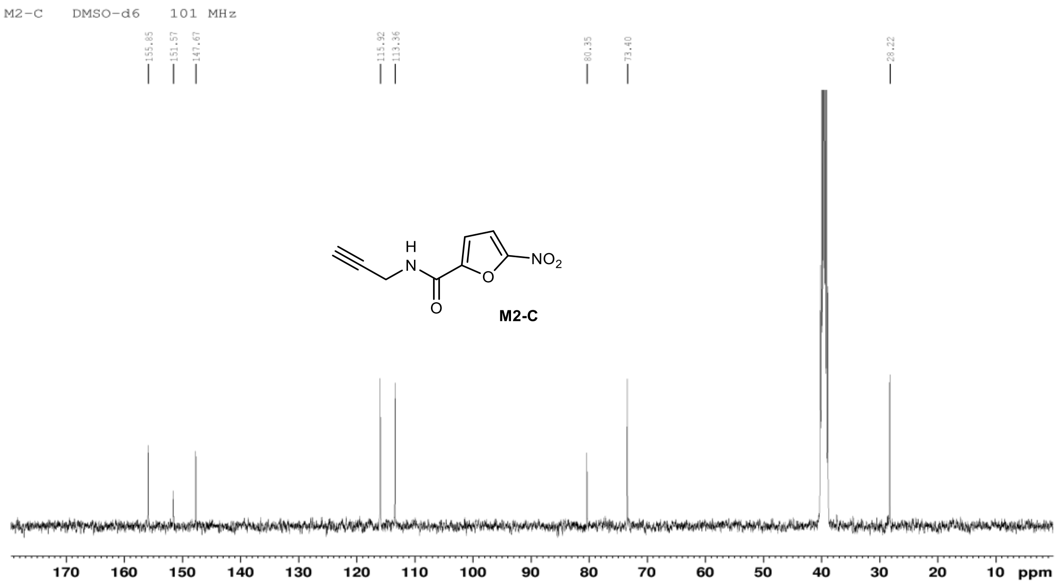

M2-C DMSO-d6 101 MHz
155.85
151.57
147.67
115.92
113.36
80.35
73.40
28.22
M2-C
170 160 150 140 130 120 110 100 90 80 70 60 50 40 30 20 10 ppm

D

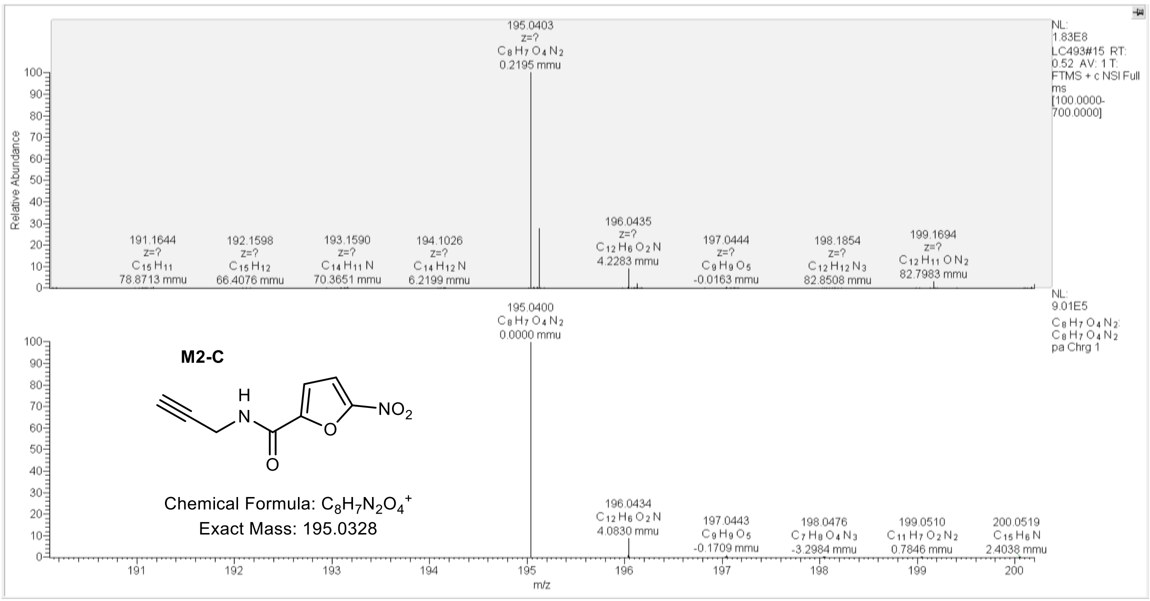

195.0403
z=?
C8 H7 O4 N2
0.2195 mmu
NL:
1.83E8
LC493#15 RT:
0.52 AV: 1 T:
FTMS + c NSI Full
ms
[100.0000-
700.0000]
Relative Abundance
191.1644
z=?
C15 H11
78.8713 mmu
192.1598
z=?
C15 H12
66.4076 mmu
193.1590
z=?
C14 H11 N
70.3651 mmu
194.1026
z=?
C14 H12 N
6.2199 mmu
196.0435
z=?
C12 H6 O2 N
4.2283 mmu
197.0444
z=?
C9 H9 O5
-0.0163 mmu
198.1854
z=?
C12 H12 N3
82.8508 mmu
199.1694
z=?
C12 H11 O N2
82.7983 mmu
NL:
9.01E5
C8 H7 O4 N2:
C8 H7 O4 N2
pa Chrg 1
195.0400
C8 H7 O4 N2
0.0000 mmu
M2-C
Chemical Formula: C8H7N2O4+
Exact Mass: 195.0328
196.0434
C12 H6 O2 N
4.0830 mmu
197.0443
C9 H9 O5
-0.1709 mmu
198.0476
C7 H8 O4 N3
-3.2984 mmu
199.0510
C11 H7 O2 N2
0.7846 mmu
200.0519
C15 H6 N
2.4038 mmu
191 192 193 194 195 196 197 198 199 200
m/z

Supplementary Figure 9. 5-Spectroscopic and spectrometric analysis of synthesized M2-C. A. Clickable M2 analog M2-C procedure synthesis. **B**. ^{1}H NMR spectrum of M2-C in dichloromethane-d2. Yield: 331 mg, 85%. Isolated as a yellowish solid, >95% pure by NMR and a single spot by TLC; R_f: 0.6 in n-Hex/EtOAc, 50/50. ^{1}H NMR (400 MHz, DMSO-*d6*) δ ppm = 9.34 (t, *J* = 5.5 Hz, 1H), 7.75 (d, *J* = 3.9 Hz, 1H), 7.44 (d, *J* = 3.9 Hz, 1H), 4.05 (dd, *J* = 2.6 Hz, *J* = 5.7 Hz, 2H), 3.18 (t, *J* = 2.5 Hz, 1H). **C**. ^{13}C NMR spectrum of M2-C in dichloromethane-d2. ^{113}C NMR (101 MHz, DMSO-*d6*) δ ppm = 155.9; 151.6; 147.7 ; 115.9 ; 113.4 ; 80.4 ; 73.4 ; 28.2. UPLC: R_T: 1.59 (classic system). **D**. HRMS spectrum of M2-C. HRMS (ESI$^+$) m/z [M+H]$^+$ Calcd for $C_8H_7N_2O_4^+$ 195,0328; Found 195.0403. Source data are provided as a Source Data file.

Supplementary Table 1. Listing of treatment dose for each cell lines and corresponding compounds.

	Compounds										
Cell lines	Nifuroxazide (NIF)	5-Nitro-2-furaldehyde (M2)	4-Hydroxydenzhydrazide (HBH)	Methyl 5-amino-2-furoate (M7)	Olaparib (PARPi)	Melphalan	Napabucasin (STAT3i)	Ruxolitinib (JAK2i)	NIF-C	M2-C	HBH-C
SW620	5µM	-	-	-	-	-	0.1µM	10µM	-	-	-
MKN45	5µM	-	-	-	-	-	-	-	-	-	-
PANC1	5µM	-	-	-	-	-	-	-	-	-	-
HT29	5µM	-	-	-	-	-	0.1µM	10µM	-	-	-
CRC1	5µM	-	-	-	-	-	-	-	-	-	-
SUM159	10µM	10µM	10µM	10µM	10µM	10µM	0.1µM	10µM	10µM	10µM	10µM
S68	10µM	10µM	10µM	10µM	10µM	10µM	0.1µM	10µM	10µM	10µM	10µM
SUM149	10µM	10µM	10µM	-	10µM	-	-	-	-	-	-
MDA-MB-436	10µM	-	-	-	-	-	-	-	-	-	-
