# Supplementary material for: Inhibition of the STAT3/Fanconi anemia axis is synthetic lethal with PARP inhibition in breast cancer
Source: Nat Commun. 2025 Mar 4;16:2159. doi: 10.1038/s41467-025-57476-4 (PMC11880418; doi:10.1038/s41467-025-57476-4)
Supplement: Supplementary file 2 — Reporting Summary [file 41467_2025_57476_MOESM2_ESM.pdf]

nature portfolio

Corresponding author(s): Emmanuelle Charafe-Jauffret
Christophe Ginestier

Last updated by author(s): Dec 20, 2024

Reporting Summary

Nature Portfolio wishes to improve the reproducibility of the work that we publish. This form provides structure for consistency and transparency in reporting. For further information on Nature Portfolio policies, see our Editorial Policies and the Editorial Policy Checklist.

Statistics

For all statistical analyses, confirm that the following items are present in the figure legend, table legend, main text, or Methods section.

n/a	Confirmed	
☐	☐	The exact sample size (*n*) for each experimental group/condition, given as a discrete number and unit of measurement
☐	☒	A statement on whether measurements were taken from distinct samples or whether the same sample was measured repeatedly
☐	☒	The statistical test(s) used AND whether they are one- or two-sided<br>*Only common tests should be described solely by name; describe more complex techniques in the Methods section.*
☐	☒	A description of all covariates tested
☐	☒	A description of any assumptions or corrections, such as tests of normality and adjustment for multiple comparisons
☐	☒	A full description of the statistical parameters including central tendency (e.g. means) or other basic estimates (e.g. regression coefficient) AND variation (e.g. standard deviation) or associated estimates of uncertainty (e.g. confidence intervals)
☐	☒	For null hypothesis testing, the test statistic (e.g. *F*, *t*, *r*) with confidence intervals, effect sizes, degrees of freedom and *P* value noted<br>*Give P values as exact values whenever suitable.*
☒	☐	For Bayesian analysis, information on the choice of priors and Markov chain Monte Carlo settings
☒	☐	For hierarchical and complex designs, identification of the appropriate level for tests and full reporting of outcomes
☒	☐	Estimates of effect sizes (e.g. Cohen's *d*, Pearson's *r*), indicating how they were calculated

Our web collection on statistics for biologists contains articles on many of the points above.

Software and code

Policy information about availability of computer code

Data collection	We access DeepCoverMOA data collection through the web interface: http://wren.hms.harvard.edu/DeepCoverMOA/#<br>We mapped all aCGH probes according to the hg19/NCBI human genome mapping database.
Data analysis	To dertermine mutation profiles we used FreeBayes version 0.9.9 for single-nucleotide variant (SNV) calling and insertions/deletion (indel) calling was done using GATK haplotype caller version 2.5-gf57256b with default parameters. For the second pipeline SNV calling was done with Mutect 1.7 and somatic indel calling with scalpel. All variants were then annotated for genes and function using ANNOVAR (version 2013-1112). In order to remove false positives, recurrent variants with no entry in public databases such as COSMIC or dbsnp were removed. Variants identified by both pipeline analyses were retained as somatic.<br><br>For all others data analysis, Graphpad Prism 5.0 was used throughout.

For manuscripts utilizing custom algorithms or software that are central to the research but not yet described in published literature, software must be made available to editors and reviewers. We strongly encourage code deposition in a community repository (e.g. GitHub). See the Nature Portfolio guidelines for submitting code & software for further information.

Data

Policy information about availability of data

All manuscripts must include a data availability statement. This statement should provide the following information, where applicable:

- Accession codes, unique identifiers, or web links for publicly available datasets
- A description of any restrictions on data availability
- For clinical datasets or third party data, please ensure that the statement adheres to our policy

In order to protect potential indirect identifiers while also supporting scientific endeavors, the data generated in this study are available upon appropriate request from the corresponding author. Email is the preferred mode of contact. Requests for non-commercial analysis should be made by researchers and include sound justification such as for use in meta-analysis. Signed data access agreements may be required.

Research involving human participants, their data, or biological material

Policy information about studies with human participants or human data. See also policy information about sex, gender (identity/presentation), and sexual orientation and race, ethnicity and racism.

Reporting on sex and gender	Breast cancer has a large female predominance and all of the PDX models were generated from patients self-identified as female. Due to biological differences including the influence of hormones, only female mice were used in the PDXs.
Reporting on race, ethnicity, or other socially relevant groupings	France do not allow patient referencing based on race, or ethnicity, or any social grouping. That being said, no formal subgroup analysis was conducted due to limited sample size.
Population characteristics	No known covariates were examined of controlled for in this study due to limited sample size
Recruitment	PDXs were selected from the living biobank previously generated (Charafe-Jauffret et al. Cancer Res, 2013; Goncalves et al., Oncotarget, 2016). We specififcally selected PDX generated from TNBCs representative of the different HRD status oberved in the breast cancer patient population.
Ethics oversight	Samples of human origin and the associated data were obtained from the IPC/CRCM Tumour Bank that operates under authorization # AC-2013-1905 granted by the French Ministry of Research. Prior to scientific use of samples and data, patients were appropriately informed and filed a written consent, in compliance with French and European regulations. The experiments were conformed to the principles set out in the WMA Declaration of Helsinki and the Department of Health and Human Services Belmont Report.

Note that full information on the approval of the study protocol must also be provided in the manuscript.

Field-specific reporting

Please select the one below that is the best fit for your research. If you are not sure, read the appropriate sections before making your selection.

☒ Life sciences ☐ Behavioural & social sciences ☐ Ecological, evolutionary & environmental sciences

For a reference copy of the document with all sections, see nature.com/documents/nr-reporting-summary-flat.pdf

Life sciences study design

All studies must disclose on these points even when the disclosure is negative.

Sample size	No sample size calculation was performed. The sample size was determined considering the variations and mean values of the samples, or based on previous observations or a standard protocol in the field.
Data exclusions	No data was excluded from analyses except for SNV calling in order to remove false positives, recurrent variants with no entry in public databases such as COSMIC or dbsnp were removed.
Replication	All biological replicates are obtained from biologically independent experiments.
Randomization	Samples/mice were randomized allocated into control and experimental groups.
Blinding	The investigators were blinded to group allocation during data collection

Reporting for specific materials, systems and methods

We require information from authors about some types of materials, experimental systems and methods used in many studies. Here, indicate whether each material, system or method listed is relevant to your study. If you are not sure if a list item applies to your research, read the appropriate section before selecting a response.

Materials & experimental systems

n/a	Involved in the study
☐	☒ Antibodies
☐	☒ Eukaryotic cell lines
☒	☐ Palaeontology and archaeology
☐	☒ Animals and other organisms
☐	☒ Clinical data
☒	☐ Dual use research of concern
☒	☐ Plants

Methods

n/a	Involved in the study
☒	☐ ChIP-seq
☐	☒ Flow cytometry
☒	☐ MRI-based neuroimaging

Antibodies

Antibodies used

anti-ALDH1A1 (mAb, Clone 44, Becton Dickinson, 1/200)
anti-CyclinD1 (rabbit mAb, Cell Signaling #55506, 1/1,000)
anti- p-STAT3 (Tyr705) (mouse mAb, Cell Signaling #4113, 1/2,000)
anti-STAT3 (mouse mAb, Cell Signaling #9139, 1/1,000)
anti-FANCD2 (rabbit mAb, abcam, ab108928, 1/1,000)
anti-FANCI (santa cruz sc-271316, 1/1,000)
anti-γH2AX (rabbit mAb, Cell Signaling #9718, 1/1,000)
anti-GAPDH (Rabbit pAb, Cell Signaling, 1/5,000)
anti-α-Actin (mouse mAb, Sigma Aldrich #A5441, 1/5,000)
anti-phospho-Histone H2AX (Ser139, clone JBW301, Merck Millipore, 1/1,000)
anti-RAD51 (gift from M. Modesti lab, CRCM, Marseille, 1/1,000)
anti-STAT3 (rabbit mAb, ab171360)
anti-H3K27ac (rabbit mAb, active motif; #39133)

Validation

All commercial antibodies have been validated by the manufacturers.
anti-RAD51 was validated by Western blot (as previously published Aldolph MB (Mol Cell, 2021)

Eukaryotic cell lines

Policy information about cell lines and Sex and Gender in Research

Cell line source(s)

HT29, SW620, MKN45 and PANC1 come from ATCC (https://www.atcc.org/). CRC1 cells were established from a CRC biopsy (CHU-Carémeau, Nîmes, France, ClinicalTrial.gov Identifier#NCT01577511) as previously reported (Planque et al., Oncotarget, 2016). SUM159 and SUM149 was given by Dr. S.Ethier (Karmanos Cancer Center, Detroit, MI, USA), S68 was given by Dr. V. Castros (Université de Rennes, France). HeLa (WT, FANCD2KO, and ERCC1KO) were given by C. Lachaud (CRCM, Marseille) and previously described in Berrada et al., NAR, 2023.

Authentication

All cell lines were not authenticated beyond inspection based on morphological criteria

Mycoplasma contamination

Cells were tested for mycoplasma contamination

Commonly misidentified lines (See ICLAC register)

no commonly misidentified cell lines were used

Animals and other research organisms

Policy information about studies involving animals; ARRIVE guidelines recommended for reporting animal research, and Sex and Gender in Research

Laboratory animals

We used (6-8 weeks) NOD.Cg-Prkdcscid Il2rgtm1Wjl/SzJ mice (Ref: 005557, Charles River). Mice were housed under sterile conditions with sterilized food and water provided ad libitum and maintained on a 12-h light and 12-h dark cycle, temperatures of 19-21°C with 40-60% humidity.

Wild animals

none

Reporting on sex

Only female mice were used as this was models of breast cancer and men account for less than 1 percent of breast cancer incidents in humans.

Field-collected samples

none

Ethics oversight

Animal studies were conducted in agreement with the French Guidelines for animal handling and approved by local ethics committee

Ethics oversight	(Agreement no. #16487-2018082108541206 v3). Of note, mouse weight loss >20%, tumor necrosis, tumor volume >1500 mm3, ruffled coat + hunched back, weakness, and reduced motility were monitored daily and considered as endpoints.

Note that full information on the approval of the study protocol must also be provided in the manuscript.

Clinical data

Policy information about clinical studies

All manuscripts should comply with the ICMJE guidelines for publication of clinical research and a completed CONSORT checklist must be included with all submissions.

Clinical trial registration	N/A
Study protocol	N/A
Data collection	N/A
Outcomes	N/A

Plants

Seed stocks	N/A
Novel plant genotypes	N/A
Authentication	N/A

Flow Cytometry

Plots

Confirm that:

☒ The axis labels state the marker and fluorochrome used (e.g. CD4-FITC).

☒ The axis scales are clearly visible. Include numbers along axes only for bottom left plot of group (a 'group' is an analysis of identical markers).

☐ All plots are contour plots with outliers or pseudocolor plots.

☒ A numerical value for number of cells or percentage (with statistics) is provided.

Methodology

Sample preparation	For cell lines, standard flow cytometry was performed on single cell suspension following cell tripsinization. Quantitative image-based cytometry was performed on nucleus that were extracted with CSK buffer (50mM NaCl, 25mM Hepes pH7.4, 3mM MgCl2, 300mM sucrose, 0.5% triton, 1mM EDTA and protease and phosphatase inhibitor) 5 minutes on ice.<br><br>For PDX, flow cytometry was performed on single cell suspension following tissue dissociation (mechanically and enzymatically using collagenase/hyaluronidase (StemCell Technologies))<br><br>For PDOx, flow cytometry was performed on single cell suspension following tissue dissociation using TrypLE Express 1X (Gibco, #12605-010) during 15 minutes at 37°C under agitation (155 RPM)
Instrument	Standard Flow cytometry was performed on LSRII cytometer (BD) and spectral cytometry Aurora cytometer (Cytek)
Software	Cytometry analysis were done with BD FACSDiva v9.0 Software
Cell population abundance	After sorting, the sorted population was at least 95% pure
Gating strategy	Gating was performed based on FSC-A (to exclude cell debris), FSC-H vs FSC-A (for single cells), and viability stain selected cells (to exclude dead cells), and used Fluorescence-minus-one (FMO) controls to determine the positive percentage expression of different surface markers. For ALDEFLUOR assay, ALDH inhibitor (DEAB) was used to determine cells presenting an ALDH enzymatic activity. For PDXs, H2Kd staining was used to gate out murine cells.

☐ Tick this box to confirm that a figure exemplifying the gating strategy is provided in the Supplementary Information.
